# KLF2 Protects against Osteoarthritis by Repressing Oxidative Response through Activation of Nrf2/ARE Signaling *In Vitro* and *In Vivo*

**DOI:** 10.1155/2019/8564681

**Published:** 2019-11-19

**Authors:** Xiang Gao, Shuangpeng Jiang, Zhangzhen Du, Angtin Ke, Qingwei Liang, Xu Li

**Affiliations:** Department of Orthopedics, The First Hospital of China Medical University, Shenyang 110000, China

## Abstract

Osteoarthritis (OA) is a multifactorial and inflammatory disease characterized by cartilage destruction that can cause disability among aging patients. There is currently no effective treatment that can arrest or reverse OA progression. Kruppel-like factor 2 (KLF2), a member of the zinc finger family, has emerged as a transcription factor involved in a wide variety of inflammatory diseases. Here, we identified that KLF2 expression is downregulated in IL-1*β*-treated human chondrocytes and OA cartilage. Genetic and pharmacological overexpression of KLF2 suppressed IL-1*β*-induced apoptosis and matrix degradation through the suppression of reactive oxygen species (ROS) production. In addition, KLF2 overexpression resulted in increased expression of heme oxygenase-1 (HO-1) and NAD(P)H dehydrogenase quinone 1 (NQO1) through the enhanced nuclear translocation of nuclear factor erythroid 2-related factor 2 (Nrf2). Further, Nrf2 inhibition abrogated the chondroprotective effects of KLF2. Safranin O/fast green and TUNEL staining demonstrated that adenovirus-mediated overexpression of KLF2 in joint cartilage protects rats against experimental OA by inhibiting cartilage degradation and chondrocyte apoptosis. Immunohistochemical staining revealed that KLF2 overexpression significantly decreases MMP13 expression caused by OA progression *in vivo*. This *in vitro* and *in vivo* study is the first to investigate the antioxidative effect and mechanisms of KLF2 in OA pathogenesis. Our results collectively provide new insights into OA pathogenesis regulated by KLF2 and a rationale for the development of effective OA intervention strategies.

## 1. Introduction

Osteoarthritis (OA) is one of the most prevalent joint diseases and is predicted to be the greatest cause of disability among patients over 40 years old by 2040 [1]. Although the occurrence and development of OA have been studied extensively, there is currently no efficient treatment that can arrest or reverse OA progression. Hence, identifying an enhanced regulatory mechanism for cartilage homeostasis in OA will help in managing this disease using specific targets. OA is caused by a cartilage homeostasis disorder that can be induced by various physiological and mechanical factors, such as age, genetics, mechanical stress, trauma, and metabolism [2–5]. A hallmark of OA is the degradation of the extracellular matrix (ECM) [4], which is considered a direct outcome of the action of matrix-degrading enzymes (MMPs). Among the MMPs, *MMP3*, *MMP9*, and *MMP13* are known to play crucial roles in OA cartilage destruction [6, 7]. The apoptosis of chondrocytes (a unique resident cell type in articular cartilage) also greatly contributes to the degradation of the ECM [8]. Because the release of MMPs and apoptosis of chondrocytes are indispensable components of OA progression, it is reasonable to hypothesize that efficient attenuation of these pathways may be a promising approach for the maintenance of cartilage integrity and homeostasis.

Oxidative stress is increasingly recognized as a component of OA pathology and is characterized by the excess generation of reactive oxygen species (ROS) [9]. Studies have demonstrated that elevated levels of ROS can promote the production of MMPs and apoptosis in chondrocytes, resulting in the destruction of the cartilage matrix in OA [9, 10]. Nuclear factor (erythroid-derived 2)-like 2 (*Nrf2*) is a key transcription factor that plays a crucial role in the protection against oxidative stress through regulating the expression of phase II antioxidant enzymes, including heme oxygenase-1 (*HO-1*) and NAD(P)H quinine oxidoreductase 1 (*NQO1*). Recently, Nrf2 activation has been identified as a major chondroprotective factor conferring protection against oxidative stress during OA progression. An *in vitro* study showed that *Nrf2* plays a pivotal role in protecting chondrocytes against IL-1*β*-induced oxidative stress and apoptosis [11]. Further, an *in vivo* study identified that cartilage damage is increased in Nrf2-knockout (KO) mouse OA models [12].

Kruppel-like factor 2 (*KLF2*), also known as lung Kruppel-like factor (LKLF), is a member of the zinc finger family. *KLF2* is a transcription factor that orchestrates the expression of a battery of genes involved in a wide variety of inflammatory disease conditions, such as rheumatoid arthritis, atherosclerosis, and chronic kidney disease [13–15]. Previous studies have demonstrated that in monocytes, KLF2 can inhibit the induction of the expression of proinflammatory factors, such as IL-1*β*, TNF-*α*, and IL-8, which also play an important role in OA pathogenesis [16]. It has also been shown that increasing KLF2 levels could be helpful in decreasing the expression of MMPs, such as MMP3 and MMP13, in monocytes, which mediate a cascade of matrix degradation processes resulting in the destruction of cartilage in OA [17]. Importantly, *KLF2* has been shown to play a crucial role in protecting against oxidative damage through the activation of the Nrf2/antioxidant-response element (ARE) signaling pathway [18, 19]. Despite these findings, the functions of KLF2 in chondrocytes and OA pathogenesis have not yet been investigated in detail, although an *in vitro* study by Yuan et al. suggested that *KLF2* regulates the degradation of COL2A1 by suppressing the IL-1*β*-induced expression of MMP13 in chondrocytes [20]. Therefore, the present study was undertaken to examine the chondroprotective effect of KLF2 and investigate its mechanisms *in vitro* and *in vivo*.

In this study, we used SW1353 human chondrocytes and rat models of OA to investigate whether KLF2 is associated with OA pathogenesis. We identified that *KLF2* expression is specifically downregulated in human OA cartilage. We then evaluated the functions of KLF2 in OA pathogenesis and examined the potential mechanisms *in vitro*. We found that pharmacological or lentiviral overexpression of KLF2 preserves cartilage homeostasis by suppressing apoptosis and MMP expression through the negative regulation of ROS. Furthermore, our data demonstrate that this chondroprotective effect of *KLF2* is mediated, at least in part, through the promotion of Nrf2 nuclear translocation. Overall, our gain-of-function (adenovirus-mediated overexpression of KLF2) approach in rat knee joint tissue clearly indicates that KLF2 is a potential therapeutic target for the treatment of osteoarthritis.

## 2. Materials and Methods

### 2.1. Human Articular Cartilage Samples

This study was reviewed and approved by the Institutional Review Board (IRB) of China Medical University. All patients involved in this study gave informed consent. Human OA cartilage samples with obvious erosion were obtained from patients undergoing total knee arthroplasty (*n* = 6). Normal cartilage samples without gross signs of degradation were obtained from patients undergoing total hip replacement for femur neck fracture (*n* = 6). Paired smooth and damaged cartilage was obtained from the same OA patients undergoing total knee arthroplasty (*n* = 10). The difference between the smooth (relatively healthy area) and damaged (severely damaged area) articular surfaces was distinguished using the Mankin scoring system [21]. Another 24 cartilage samples were obtained from 24 patients with different degrees of OA according to the Kellgren-Lawrence scale system (*n* = 6 per group). After surgical harvesting, the cartilage samples were immediately stored at -80°C for mRNA extraction or fixed in 4% paraformaldehyde for histological and immunohistochemical analyses.

### 2.2. SW1353 Human Chondrocyte Culture and Treatment

SW1353 human chondrocytes (American Type Culture Collection, Manassas, VA, USA) were seeded on 6-well plates and cultured in DMEM/high-glucose (HyClone, Logan, UT) containing 10% FBS (HyClone) and 1% penicillin/streptomycin (Solarbio, Beijing, China) at 37°C in humidified air with 5% CO_2_, and the medium was replaced every 2 days. At approximately 80% confluence, the cells were serum starved overnight and then stimulated with 20 ng/ml IL-1*β* (R&D Systems), 10 *μ*M simvastatin (Calbiochem), 10 *μ*M GGPP (Sigma), and 5 *μ*M ML385 (MCE) when appropriate. The concentrations were derived from previously published articles [11, 22–24].

### 2.3. Cell Viability Assay

Cell viability was determined with a cell counting kit-8 (CCK-8; Beyotime Biotechnology) according to the manufacturer's instructions. In brief, SW1353 cells were plated in 96-well plates at a density of 2000 cells/well. At the appropriate time point, 10 *μ*l of CCK-8 solution was added to each well and the plate was incubated at 37°C for 2 h. Subsequently, the absorbance of each well at 450 nm was measured.

### 2.4. Real-Time Reverse Transcription-Polymerase Chain Reaction

After the appropriate treatment, total RNA was isolated from SW1353 cells or cartilage tissues using TRIzol reagent (Life Technologies Corporation, USA) following the manufacturer's instructions. Single-stranded cDNA was synthesized from purified RNA using a RevertAid first-strand cDNA synthesis kit (Thermo Fisher Scientific, USA) in accordance with the manufacturer's instructions. cDNA was used for real-time PCR analysis using a SYBR® Premix Ex TaqTM kit (Takara Bio, China) on an ABI 7500 Fast Real-Time PCR system (Applied Biosystems, USA) according to the manufacturer's instructions. Primers were synthesized by Sangon Biotech (China). All samples were analyzed in triplicate. The mRNA value for the target gene was determined using the 2^−*ΔΔ*Ct^ method.

### 2.5. Western Immunoblotting and Immunoprecipitation

After receiving the indicated treatments, SW1353 cells were washed with cold phosphate-buffered saline (PBS). Total proteins were extracted using RIPA lysis buffer (Beyotime) with 1 mM phenylmethanesulfonyl fluoride (PMSF, Beyotime). Cytosolic and nuclear proteins were extracted by a Nuclear and Cytoplasmic Protein Extraction kit (Beyotime) according to the manufacturer's instructions. Protein concentrations were calculated using a BCA Protein Assay kit (Beyotime). Samples containing equivalent amounts of lysate protein (20 *μ*g) were separated on sodium dodecyl sulfate-polyacrylamide gels (SDS-PAGE) and transferred to a PVDF membrane (Bio-Rad, USA). After blocking in 5% nonfat milk for 2 h at room temperature, the membranes were incubated with primary antibodies in TBST overnight at 4°C. Antibodies against KLF2 were purchased from Abcam. Antibodies against MMP3, MMP9, MMP13, COL2A1, Nrf2, HO-1, NQO1, and *β*-actin were purchased from Proteintech. Thereafter, the membranes were washed with TBST and then incubated with a secondary antibody (HRP-conjugated AffiniPure goat anti-rabbit IgG, Proteintech) for 2 h at room temperature. The blots were visualized by enhanced chemiluminescence (ECL, Beyotime). The densities of the protein bands were quantified using ImageJ (http://rsb.info.nih.gov/ij/, Bethesda, MD, USA). For immunoprecipitation, cells were overexpressed with KLF2 using lentivirus. After that, the cells were rinsed with phosphate-buffered saline, scraped into lysis buffer (150 mM NaCl, 50 mM Tris-HCl at pH 7.5, 5 mM EDTA, 1 mM phenylmethylsulfonyl fluoride, and 0.5% Nonidet P-40), and placed on ice for 30 min. Cells were then spun at 15,000g for 15 min, and the supernatant was incubated overnight at 4°C with either anti-KLF2, anti-Nrf2 antibody, or control IgG at 4°C and protein A/G agarose resin for overnight. The samples were washed in lysis buffer for five times and then were used for immunoblot analysis.

### 2.6. Immunofluorescence Staining

After receiving the indicated treatments, the SW1353 cells were washed twice with PBS and fixed in 4% formaldehyde for 15 min. After being washed three times with PBS, the cells were treated with 0.2% Triton X-100 at 37°C for 20 min. The cells were then washed three times with PBS (5 min each) and blocked with 5% bovine serum albumin (BSA) at 37°C for 1 h. After washing with PBS, KLF2 (Abcam), Nrf2 (Proteintech), and MMP13 (Proteintech) antibodies (1 : 200) were added for overnight incubation at 4°C. An Alexa Fluor 488 or 594-labeled secondary antibody (1 : 200) (Proteintech) was then added for 30 min incubation at 37°C. Following washing with PBS, 0.1% DAPI (Beyotime) was added for 10 min of staining. The slides were observed using fluorescence microscopy (Olympus).

### 2.7. Measurement of Reactive Oxygen Species (ROS) Levels

After cells received the indicated treatments, the level of intracellular ROS was measured using a Reactive Oxygen Species Assay kit (Beyotime). Briefly, SW1353 cells were harvested and stained with 10 *μ*M DCF-DA in the dark at 37°C for 15 min. The cells were then rinsed 3 times with PBS and immediately detected using a flow cytometer (525 nm).

### 2.8. Measurement of Apoptosis

After receiving the indicated treatments, the SW1353 cells were collected and suspended in binding buffer. Then, the cells were stained with Annexin V-FITC/propidium iodide (PI) for 15 min in the dark according to the manufacturer's instructions (Becton Dickinson). Stained cells were detected by flow cytometry and analyzed using FlowJo software. Apoptosis of chondrocytes in rat cartilage tissue was detected using a terminal deoxynucleotidyl transferase dUTP nick-end labeling (TUNEL) assay kit (Beyotime) according to the manufacturer's instructions.

### 2.9. Lentivirus-Mediated Overexpression of KLF2

Human KLF2 and nonspecific control lentiviruses were purchased from Genechem (Shanghai, China). SW1353 cells were transfected with lentivirus in accordance with the manufacturer's instructions. KLF2 overexpression was confirmed by real-time PCR and Western blotting.

### 2.10. Animal OA Model and Adenovirus-Mediated Overexpression of KLF2 in Rat Knee Joint Tissue

Male Sprague-Dawley rats (8 weeks old, 210 ± 10 g in weigh, and specifically pathogen-free) were used in the present study. All rats were housed with a 12 h light/dark cycle at a constant room temperature (25°C) with free access to food and water. Experimental OA was induced in 8-week-old male rats by intra-articular (IA) injection of monoiodoacetate (MIA; 1 mg per cavity in 50 *μ*l of sterile saline). The control group received an IA injection of 50 *μ*l of sterile saline. Adenovirus-*KLF2* (Ad-*KLF2*) and a control virus (Ad-C) were used to overexpress KLF2 in the rat knee joint. After injection of MIA or sterile saline on day 3, Ad-KLF2 or Ad-C was injected into the knee joints of rats for three consecutive weeks (1 × 10^9^ plaque-forming units (PFUs) in a total volume of 10 *μ*l), as indicated in a previous study [25]. ML385 was used to inhibit Nrf2 expression in the rat knee joint. After the first IA injection of Ad-KLF2 on day 3, rat received an IA injection of ML385 (10 *μ*M, 20 *μ*l per joint) two times a week for 4 weeks. Rats were sacrificed 6 weeks after injection of MIA or sterile saline and subjected to histological analyses.

### 2.11. Histopathologic Analysis

Human knee articular samples and rat knee joints were prepared and fixed in 4% paraformaldehyde. Then, the samples were decalcified in 10% EDTA for 21 days and embedded in paraffin. Tissue sections (5 *μ*m) were stained with safranin O/fast green following standard protocols to determine cartilage degradation under light microscopic examination. The Osteoarthritis Research Society International (OARSI) scoring system was used to assess joint cartilage degeneration [26]. Because both the tibial and femoral cartilages were assessed in the present study, the maximum OARSI score was 48. Three independent investigators who were blinded to the experimental groups performed the scoring. Immunohistochemistry was further performed to analyze the protein expression of KLF2, MMP13, and COL2A1 in histological sections of human knee articular samples and rat knee joints. Primary antibodies against KLF2 (Abcam), MMP13, and COL2A1 (Proteintech) were used at 1 : 100-1 : 200 dilutions and incubated overnight at 4°C. Then, the sections were incubated with a biotinylated secondary antibody. The reaction was developed using a DAB kit (BD Bioscience, Franklin Lakes, NJ, USA). The expression of KLF2 and MMP13 was evaluated by calculating the percentage of immunopositive cells. The expression of COL2A1 was evaluated by calculating the relative intensity.

### 2.12. Statistical Analysis

Each experiment was repeated three times using three independent samples, and the data were expressed as the mean ± standard deviation. The *n*-value indicates the number of human specimens or rats per group. All statistical analyses were performed using GraphPad Prism software. For cell-based *in vitro* studies, statistical significance was analyzed by means of unpaired Student's *t*-test for comparisons between two groups and one-way ANOVA with post hoc test for multiple comparisons. For rat-based *in vivo* studies, the nonparametric Mann-Whitney *U* test was used. The Pearson *χ*^2^ test was used to analyze the relationship between KLF2 and COL2A1 expression levels. A *p* value < 0.05 was considered statistically significant.

## 3. Results

### 3.1. KLF2 Expression Is Downregulated in Human OA Cartilage

**Figure 1:**
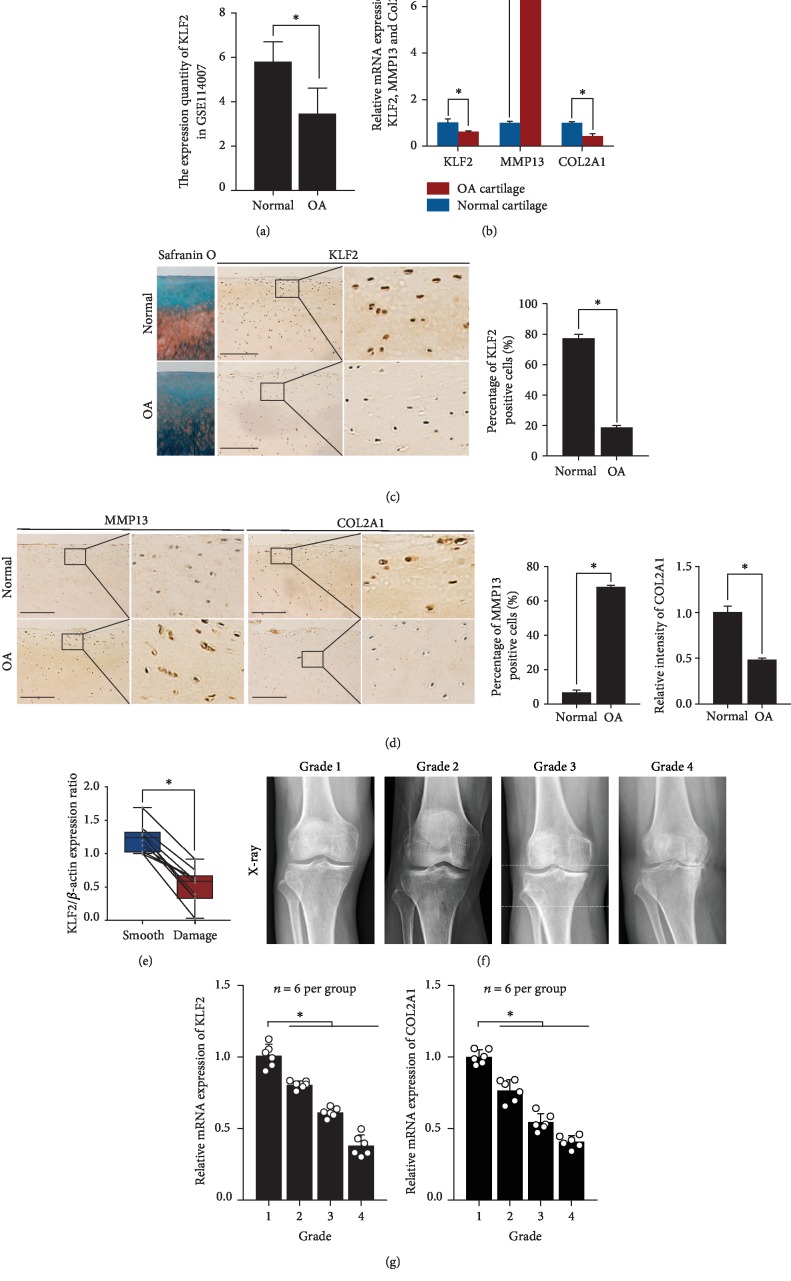
KLF2 expression is suppressed during osteoarthritis pathogenesis. (a) Microarray analysis of KLF2, MMP13, and COL2A1 expression in OA cartilage (*n* = 20) and normal cartilage (*n* = 18) based on GSE114007. (b) Real-time PCR analysis was used to assess the mRNA levels of KLF2 in normal and OA cartilage from human patients (*n* = 6 per group). (c) Representative images of safranin O/fast green staining and immunohistochemical staining with antibodies against KLF2 in normal and OA cartilage from human knee joints (*n* = 6 per group). The scale bar represents 200 *μ*m. The right panels show the quantification of KLF2 immunohistochemical staining. (d) Representative images of immunohistochemical staining with antibodies against MMP13 and COL2A1 in normal and OA cartilage from human knee joints (*n* = 6 per group). The scale bar represents 200 *μ*m. The right panels show the quantification of the immunohistochemical staining of MMP13 and COL2A1. (e) RNA was isolated from smooth and damaged regions of OA cartilage from the same patient (*n* = 10), and the levels of KLF2 mRNA were evaluated by real-time PCR. (f) Representative X-rays of different stages of OA graded by the Kellgren-Lawrence scale system. (g) RNA was isolated from grade 1-4 OA patients, and KLF2 mRNA expression was evaluated by real-time PCR (*n* = 6 per group). All data are expressed as the mean ± SD. ^∗^*p* < 0.005 and ^∗∗^*p* < 0.0005.

To assess the possible association of KLF2 with OA pathogenesis, we first downloaded (https://www.ncbi.nlm.nih.gov/gds/) and analyzed the GSE114007 microarray data, which include 18 normal and 20 OA human knee cartilage tissues. The cartilage tissues in GSE114007 were obtained from the weight-bearing regions on the medial and lateral femoral condyles. The results from the microarray analysis showed that KLF2 expression was obviously decreased in OA cartilage tissues compared to normal cartilage tissues (Figure 1(a)). To further validate this finding, we collected 6 pairs of OA cartilage tissues from patients undergoing total knee arthroplasty and normal cartilage tissues from patients with femoral neck fractures. Real-time PCR was used to detect the mRNA expression of KLF2, MMP13, and COL2A1. The results indicated that compared to that in normal cartilage tissues, KLF2 expression in OA cartilage tissues was decreased (Figure 1(b)). As expected, the expression of the osteoarthritic marker MMP13 was significantly increased, and COL2A1 expression was significantly reduced in articular cartilage in the OA samples (Figure 1(b)). Histologic changes between normal and OA samples were explored by safranin O/fast green staining. Severe cartilage loss was observed in OA cartilage tissues (reduced safranin O staining) (Figure 1(c)). Consistent with the mRNA level results, the immunostaining results revealed that compared to normal cartilage tissues, OA cartilage tissues displayed decreased expression of KLF2 and COL2A1 and high expression levels of MMP13, reflecting cartilage degradation (Figures 1(c) and 1(d)). To further support the above results, we next explored the expression pattern of KLF2 in the smooth and damaged cartilage samples from the same OA patient. The mRNA expression of KLF2 in the damaged cartilage tissue was considerably decreased compared with that in the smooth cartilage tissue from the same patient (Figure 1(e)). We next explored the correlation between the expression levels of KLF2 and the severity of knee OA. The severity of knee OA was determined by the Kellgren-Lawrence scale system [27], which is a common method of classifying the severity of knee OA using five grades (0-4) (Figure 1(f)). The expression levels of KLF2 and COL2A1 were detected using real-time PCR. Our results showed that both KLF2 and COL2A1 mRNA levels gradually decreased as the grade decreased from grade 1 to grade 4 (Figure 1(g)). Pearson's correlation analysis was used to explore the correlation between KLF2 and COL2A1 expression levels. The results indicated a significant correlation between the expression level of KLF2 and COL2A1 (Pearson's *R* value = 0.8647, *p* < 0.05). These clinical results collectively indicate that KLF2 may be involved in the pathogenesis of OA.

### 3.2. KLF2 Expression Is Downregulated in SW1353 Human Chondrocytes during IL-1*β*-Induced OA Pathogenesis

**Figure 2:**
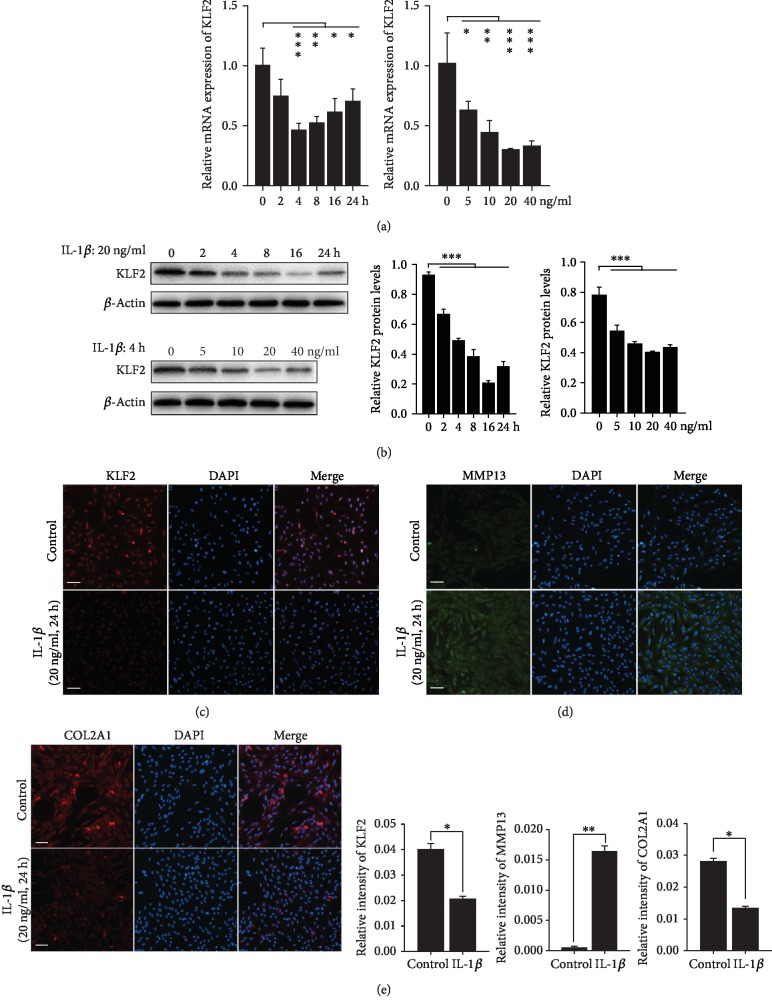
Expression of KLF2 in SW1353 cells stimulated with IL-1*β* at different concentrations for various durations. (a, b) SW1353 cells were pretreated with IL-1*β* (20 ng/ml) for the indicated times or with IL-1*β* (4 h) at the indicated concentration. KLF2 expression was measured by real-time PCR (a) and Western blot analysis (b). *β*-Actin was used as an endogenous control. Quantitative analysis of KLF2 protein levels based on the specific signal intensities measured using ImageJ. (c–e) Detection of KLF2, MMP13, and COL2A1 expression in SW1353 cells treated with IL-1*β* (20 ng/ml, 224) by immunofluorescence microscopy. The scale bar represents 50 *μ*m. All data are expressed as the mean ± SD. ^∗^*p* < 0.05, ^∗∗^*p* < 0.005, and ^∗∗∗^*p* < 0.0005.

Accumulating evidence indicates that interleukin-1*β* (IL-1*β*) is a proinflammatory cytokine that plays a critical role in the pathogenesis of OA [28, 29]. To test the influence of proinflammatory cytokines on the expression of KLF2, we examined the effect of IL-1*β* on the expression of KLF2 in SW1353 human chondrocytes by real-time PCR and Western blot analysis. IL-1*β* caused dose- and time-dependent decreases in KLF2 mRNA and protein levels in SW1353 human chondrocytes (Figures 2(a) and 2(b)). We also measured KLF2 protein levels by immunofluorescence staining, which indicated decreased KLF2 expression in IL-1*β*-treated cells (20 ng/ml, 24 h) and confirmed that KLF2 was localized in the nuclei of SW1353 cells (Figure 2(c)). The immunofluorescence staining results also showed that IL-1*β* treatment significantly decreased COL2A1 protein expression levels and increased MMP13 protein expression levels in chondrocytes, which indicates that IL-1*β* stimulation successfully mimics OA pathogenesis *in vitro* (Figures 2(d) and 2(e)). Altogether, these results further confirm that KLF2 may be involved in the pathogenesis of OA *in vitro*.

### 3.3. KLF2 Protects SW1353 Cells from IL-1*β*-Induced Apoptosis and Matrix Degradation through the Suppression of ROS Production

**Figure 3:**
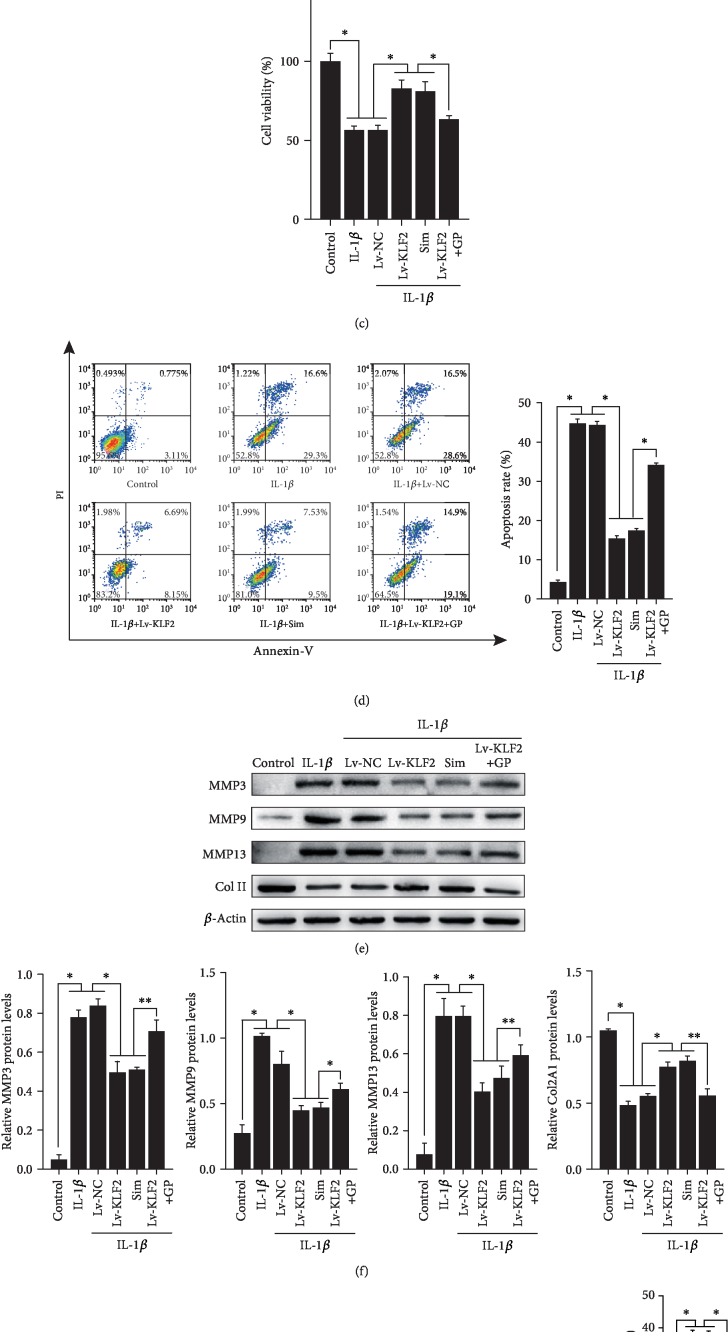
KLF2 overexpression inhibits IL-1*β*-induced apoptosis and chondrocyte degradation through the suppression of ROS production *in vitro*. SW1353 cells were infected with nonspecific control virus (Lv-NC) or KLF2-expressing virus (Lv-KLF2) or incubated with the pharmacological KLF2 activator simvastatin (Sim). After 24 h, cells were treated with or without GGPP (GP, an inhibitor of KLF2) for 8 h prior to 24 h treatment with 20 ng/ml IL-1*β*. (a) Real-time PCR was performed to assess KLF2 mRNA expression levels. (b) Western blotting analysis was performed to assess KLF2 expression at the protein level. *β*-Actin was used as an endogenous control. Quantitative analysis of KLF2 protein levels based on the specific signal intensities measured using ImageJ. (c) Cell viability was determined by CCK-8 assay. (d) Apoptosis was determined by Annexin V/PI staining followed by flow cytometry assays. (e) Expression of MMP3, MMP9, MMP13, and COL2A1 was investigated by Western blotting analysis. *β*-Actin was used as an endogenous control. (f) Quantitative analysis of MMP3, MMP9, MMP13, and COL2A1 protein levels based on specific signal intensities measured using ImageJ. SW1353 cells were infected with Lv-NC or Lv-KLF2 or incubated with the pharmacological activator of KLF2 simvastatin. After 24 h, cells were treated with or without GP for 8 h prior to 30 min treatment with 10 ng/ml IL-1*β*. (g) ROS generation was determined by DCF-DA staining followed by flow cytometry analysis. All data are expressed as the mean ± SD. ^∗^*p* < 0.005 and ^∗∗^*p* < 0.0005.

Since apoptosis of chondrocytes is considered a central feature in OA progression [30], we investigated the effects of KLF2 overexpression on IL-1*β*-induced apoptosis in SW1353 cells using pharmacological and lentiviral strategies. As shown in Figures 3(a) and 3(b), KLF2 expression was significantly increased at the mRNA and protein levels by specific KLF2 overexpression using lentivirus or treatment with the KLF2 activator simvastatin [31], whereas it was significantly decreased by treatment with the KLF2 inhibitor GGPP. Our CCK-8 results showed that IL-1*β* treatment significantly decreased SW1353 cell viability, whereas genetic or pharmacological overexpression of KLF2 rescued the IL-1*β*-induced decrease in cell viability (Figure 3(c)). To further explore the protective role of KLF2, KLF2-overexpressing cells were treated with the KLF2-specific inhibitor GGPP. As shown in Figure 3(c), GGPP abrogated the effects of KLF2 overexpression on cell viability. Consistent with these findings, genetic or pharmacological overexpression of KLF2 significantly decreased the percentage of IL-1*β*-induced apoptotic SW1353 cells, while the addition of the KLF2 inhibitor GGPP abrogated the effects of KLF2 overexpression on SW1353 apoptosis (Figure 3(d)).

Since the degradation of articular cartilage is a hallmark of OA pathogenesis, we next explored the effects of KLF2 overexpression on IL-1*β*-induced cartilage degradation in SW1353 cells. We measured the expression of MMP3, MMP9, MMP13, and COL2A1 (the primary component of ECM) in SW1353 cells with and without KLF2 overexpression and stimulated with IL-1*β*. Our results indicated that genetic or pharmacological overexpression of KLF2 in conjunction with IL-1*β* stimulation significantly reduced MMP3, MMP9, and MMP13 expression and increased COL2A1 expression in SW1353 cells; furthermore, the addition of the KLF2 inhibitor GGPP abrogated the effects of KLF2 overexpression (Figures 3(e) and 3(f)).

Oxidative stress is increasingly recognized as being closely associated with OA pathology, and accumulating evidence has demonstrated that elevated levels of ROS lead to apoptosis and ECM degradation in OA pathogenesis [5]. To determine whether the chondroprotective effects of KLF2 overexpression are due to attenuated ROS production, we monitored the extent of ROS production in SW1353 cells. Our results showed that IL-1*β* stimulation significantly increased the levels of ROS in SW1353 cells, whereas genetic or pharmacological overexpression of KLF2 in SW1353 cells suppressed the IL-1*β*-induced generation of ROS (Figure 3(g)). This effect was partially weakened by the addition of the KLF2 inhibitor GGPP (Figure 3(g)). These results collectively indicate that the overexpression of KLF2 effectively alleviates IL-1*β*-induced catabolic events and apoptosis through the suppression of ROS generation in chondrocytes.

### 3.4. KLF2 Overexpression Inhibits Matrix Degradation and SW1353 Cell Apoptosis through the Activation of the Nrf2/ARE Signaling Pathway

**Figure 4:**
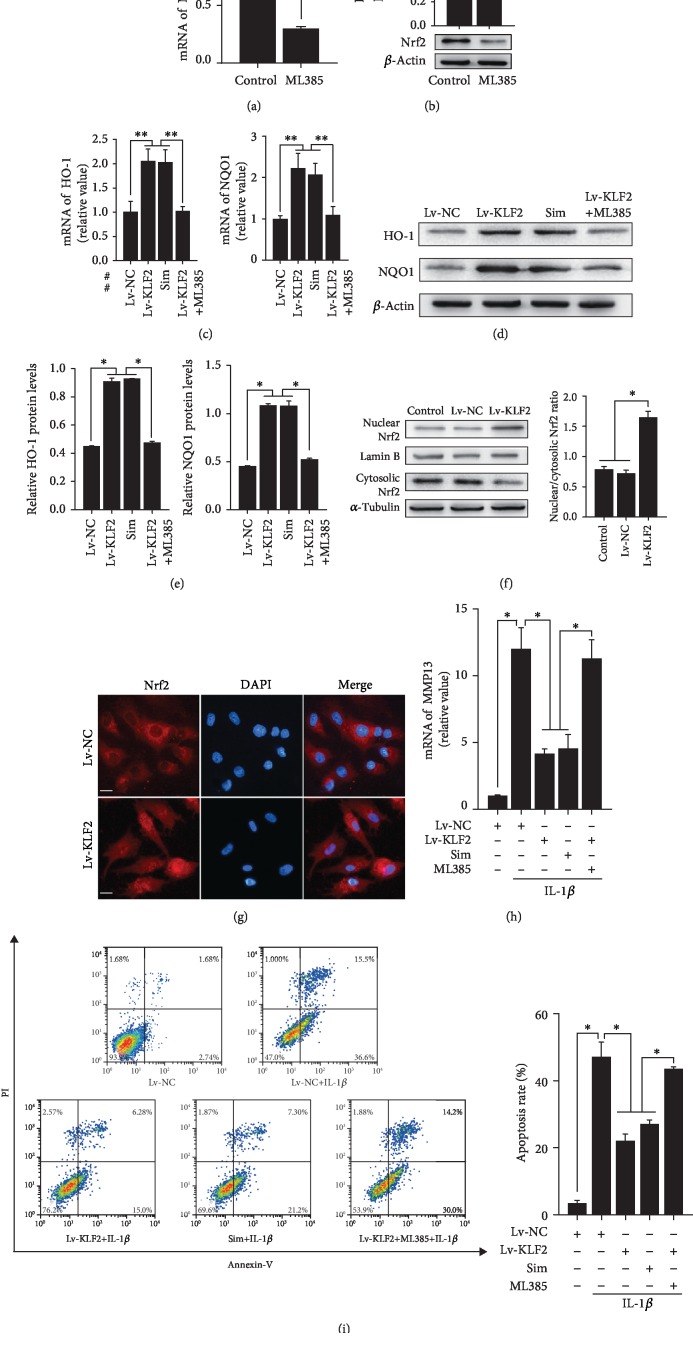
KLF2 exerts chondroprotective effects through the induction of Nrf2 nuclear translocation. (a, b) SW1353 cells were treated with ML385 (an inhibitor of Nrf2). After 12 h, Nrf2 expression was evaluated by real-time PCR (a) and Western blotting analysis (b). *β*-Actin was used as an endogenous control. Quantitative analysis of Nrf2 protein levels based on specific signal intensities measured using Image. (c–e) SW1353 cells were infected with nonspecific control virus (Lv-NC) or a KLF2-expressing virus (Lv-KLF2) or incubated with the pharmacological activator of KLF2 simvastatin (Sim). After 24 h, cells were treated with or without ML385 for 12 h. HO-1 and NQO1 mRNA levels were evaluated by real-time PCR (c), and protein levels were evaluated by Western blot analysis (d). *β*-Actin was used as an endogenous control. Quantitative analysis of HO-1 and NQO1 protein levels based on specific signal intensities measured using ImageJ (e). (f, g) SW1353 cells were infected with Lv-NC or Lv-KLF2. Nrf2 protein levels in the cytosolic and nuclear fractions were evaluated by Western blotting analysis (f). *α*-Tubulin (cytosolic) and Lamin B (nuclear) were used as endogenous controls. Quantitative analysis of Nrf2 protein levels based on specific signal intensities measured using ImageJ. (g) Immunohistochemical staining of the subcellular localization of Nrf2 protein (red). Nuclei were stained with DAPI (blue). The scale bars represent 10 *μ*m. (h, i) SW1353 cells were infected with Lv-NC or Lv-KLF2 or incubated with simvastatin. After 24 h, cells were treated with or without ML385 for 12 h prior to 24 h of treatment with 20 ng/ml IL-1*β*. MMP13 mRNA levels were evaluated by real-time PCR (h), and apoptosis was determined by Annexin V/PI staining followed by flow cytometry (i). All data are expressed as the mean ± SD. ^∗^*p* < 0.005 and ^∗∗^*p* < 0.0005.

The Nrf2/ARE signaling pathway is primarily responsible for cellular defences against oxidative stress under the pathological conditions of OA [11]. To further explore the underlying mechanism of the KLF2-mediated suppression of IL-1*β*-induced ROS generation in SW1353 cells, we determined whether the chondroprotective effect of KLF2 is mediated by the activation of the transcription factor Nrf2. As shown in Figures 4(a) and 4(b), the expression of Nrf2 in SW1353 cells was successfully ablated by ML385 (an inhibitor of Nrf2) treatment. We then examined the effect of KLF2 overexpression on the expression of Nrf2 downstream proteins in SW1353 cells. The results showed that overexpression of KLF2 caused a significant increase in NQO1 and HO-1 expression at the mRNA and protein levels, while the addition of the Nrf2 inhibitor ML385 abrogated the KLF2-induced increase in HO-1 and NQO1 expression (Figures 4(c)–4(e)). To study how KLF2 affects Nrf2 activity during oxidative stress, SW1353 cells were infected with nonspecific control lentiviruses or KLF2 lentiviruses. Western blot analysis of Nrf2 revealed that KLF2 promotes the nuclear translocation of Nrf2 protein in SW1353 cells (Figure 4(f)). Similar results were observed in a parallel immunofluorescence analysis (Figure 4(g)). This observation is consistent with those of previously published reports in endothelial cells and hepatic stellate cells [18, 23]. To determine whether KLF2 could interact with Nrf2 in cells, immunoprecipitation was performed. However, the results showed that the protein-protein interaction between KLF2 and Nrf2 was undetected in vitro (Fig. [Supplementary-material supplementary-material-1]). It is likely that KLF2-mediated promotion of Nrf2 nuclear translocation is indirectly enhanced by other mechanisms rather than interacting with each other at the protein level in human articular chondrocytes. To further demonstrate that the chondroprotective effect of KLF2 is Nrf2-dependent, we inhibited Nrf2 expression using ML385. The results showed that Nrf2 inhibition significantly abrogated the KLF2-mediated suppression of MMP13 and apoptosis induced by IL-1*β* in SW1353 cells (Figures 4(h) and 4(i)). Taken together, these results suggest that the protective effects of KLF2 overexpression in OA are associated with the regulation of the Nrf2/ARE signaling pathway *in vitro*.

### 3.5. KLF2 Protects against MIA-Induced Cartilage Destruction in a Rat OA Model

**Figure 5:**
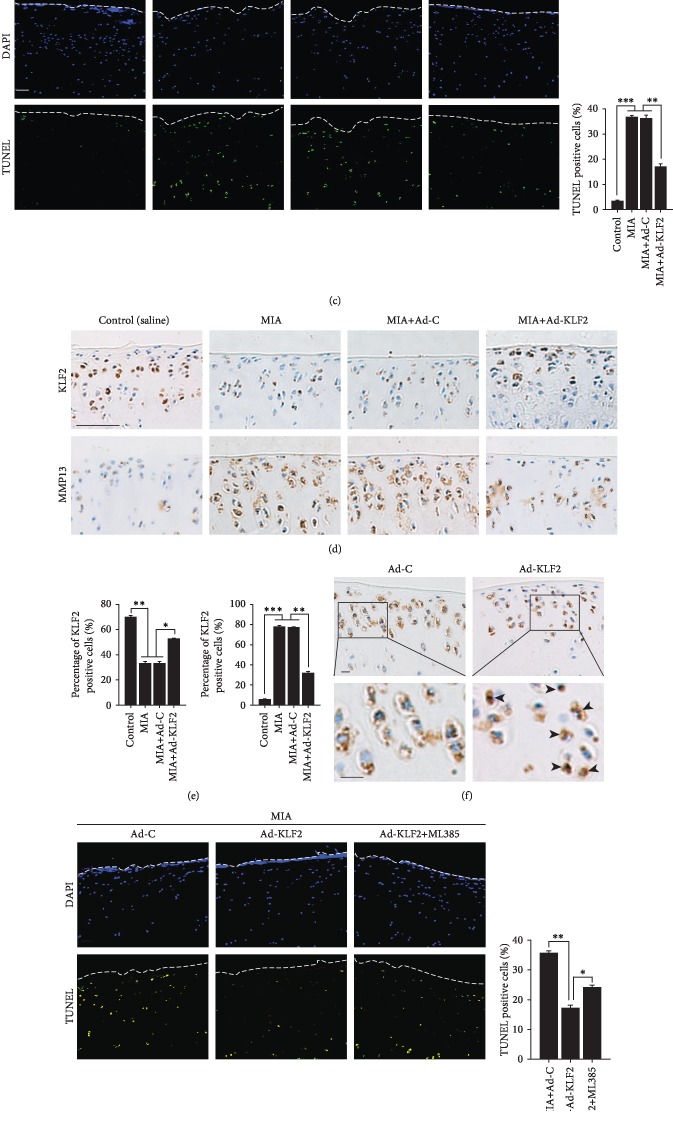
KLF2 protects against MIA-induced osteoarthritis *in vivo*. (a) Schematic depiction of the time of treatment. (b) Rats subjected to IA injection with sterile saline or MIA were IA-injected with Ad-C as a control or Ad-KLF2 to overexpress KLF2 in cartilage tissues (*n* = 11 per group). Cartilage destruction was determined by safranin O staining and evaluated by OARSI grade. The scale bar represents 200 *μ*m. (c) Detection and quantitation of apoptotic chondrocytes in cartilage by TUNEL staining. The scale bar represents 100 *μ*m. (d) Expression of KLF2 and MMP13 was evaluated by immunohistochemical staining. The scale bar represents 50 *μ*m. (e) Quantification of the immunohistochemical staining of KLF2 and MMP13. (f) Expression of Nrf2 was evaluated by immunohistochemical staining. Black arrowheads indicate the cells with markedly increased expression levels of nuclear Nrf2. The scale bar represents 10 *μ*m. (g) Rats subjected to IA injection with MIA were IA-injected with Ad-C as a control or Ad-KLF2 to overexpress KLF2 in cartilage tissues with or without IA injection of ML385 (*n* = 11 per group). Detection and quantitation of apoptotic chondrocytes in cartilage by TUNEL staining. The scale bar represents 100 *μ*m. (h) Expression of MMP13 was evaluated by immunohistochemical staining. The scale bar represents 50 *μ*m. All data are expressed as the mean ± SD. ^∗^*p* < 0.05, ^∗∗^*p* < 0.005, and ^∗∗∗^*p* < 0.0005.

The potential effects of KLF2 upregulation as a new therapeutic strategy were analyzed in OA animals (Figure 5(a)). We overexpressed KLF2 in knee joint cartilage tissues of 8-week-old male rats via IA injection of an adenovirus expressing KLF2 (Ad-*KLF2*). Previous studies have demonstrated that an adenovirus system effectively delivers genes to knee joint cartilage tissues [25, 32]. KLF2 overexpression significantly decreased the cartilage damage caused by MIA injection, as determined by safranin O staining and OARSI grade scoring (Figure 5(b)). The TUNEL staining revealed that KLF2 overexpression significantly decreased the percentage of apoptotic chondrocytes caused by MIA injection (Figure 5(c)). In addition, the results from immunohistochemical staining revealed that KLF2 protein levels were significantly decreased in the MIA group compared to those in the control group (Figures 5(d) and 5(e)). By comparing the KLF2 expression levels in the MIA+Ad-C group and the MIA+Ad-KLF2 group, we also identified the successful upregulation of KLF2 expression by IA injection of Ad-KLF2 (Figures 5(d) and 5(e)). We next examined the effects of KLF2 on the expression of MMP13 *in vivo*. The results showed that KLF2 overexpression significantly decreased MMP13 expression caused by MIA injection (Figures 5(d) and 5(e)). Furthermore, immunohistochemical staining revealed that KLF2 overexpression effectively promoted the nuclear translocation of Nrf2 *in vivo* (Figure 5(f)). To further demonstrate that the therapeutic effect of KLF2 is partly Nrf2-dependent, we inhibited Nrf2 expression using ML385 through IA injection. The results showed that Nrf2 inhibition significantly abrogated the KLF2-mediated suppression of apoptosis and MMP13 induced by MIA in *vivo* (Figures 5(g) and 5(h)). These results indicate that KLF2 acts as a catabolic regulator of cartilage degeneration and OA pathogenesis and that KLF2 could be a potential therapeutic target for OA.

## 4. Discussion

Although several factors, such as age, genetic heritage, mechanical stress, trauma, and metabolism, are associated with OA progression, the alterations in cell signaling and metabolism that occur in chondrocytes are not yet fully known. In the present study, we uncovered that KLF2 expression is decreased during OA progression and that the modulation of KLF2 levels alters cartilage homeostasis by regulating apoptosis and ECM degradation through the regulation of ROS production. Mechanistically, we found that KLF2 overexpression promotes the nuclear translocation of Nrf2. We then further employed OA animal models to verify the potential therapeutic effect of KLF2 in OA progression.

KLFs are zinc finger family members, which serve as critical transcription factors in various biological processes [33]. Among the family members, KLF2 has been the most widely studied for its role in the activation of immune cells and the regulation of inflammation [34]. To date, two studies have reported the pathological and biological role of KLF2 in rheumatoid arthritis (RA). One study reported that the upregulation of KLF2 is associated with the downregulation of MMPs in monocytes [17]. Another study showed that KLF2 has the potential to attenuate the inflammatory properties of monocytes and regulate monocyte differentiation in the context of RA [13]. Although RA and OA share many similar features of joint damage, they represent different etiological and pathological processes. Whether KLF2 regulates cartilage homeostasis in OA pathogenesis has not been investigated in detail. The only study to date describing a role for KLF2 in chondrocytes is the *in vitro* study by Yuan et al., who reported that KLF2 regulates the degradation of COL2A1 by suppressing the IL-1*β*-induced expression of MMP13 [20]. A relatively large number of studies have reported that KLF2 expression is consistently reduced across a diverse array of acute or chronic inflammatory conditions [13, 16, 17, 35]. Consistent with these previous reports, our initial analysis using microarray (GSE114007) and clinical cartilage samples revealed that KLF2 is downregulated in human OA cartilage. In addition, our data suggest for the first time that KLF2 expression levels are significantly correlated with OA severity.

Considering this correlation between KLF2 expression and OA conditions, we next evaluated the possible protective effects of KLF2 *in vitro* under IL-1*β* stimulation conditions. IL-1*β* is a critical proinflammatory cytokine in the pathogenesis of OA and is usually utilized to stimulate chondrocytes to mimic the OA conditions in *in vitro* studies [11, 20, 29]. In the present study, KLF2 expression was downregulated in cells stimulated with IL-1*β* in a dose- and time-dependent manner. Genetic or pharmacological overexpression of KLF2 in chondrocytes stimulated with IL-1*β* inhibited the expression of MMPs, including MMP3, MMP9, and MMP13, and increased the production of COL2A1. Pharmacological inhibition of KLF2 using GGPP resulted in a significant blockade of the KLF2 overexpression-induced effects on MMPs and COL2A1. These observations indicate that KLF2 functions as a critical regulator of MMPs and COL2A1, which are both crucial molecules in the OA-related degradation of the ECM [6, 7]. To the best of our knowledge, chondrocyte apoptosis is an underlying factor for the initiation of OA [30, 36]. Considering the antiapoptotic properties of KLF2 [37, 38], we performed flow cytometry experiments to detect changes in apoptosis. The results indicated that genetic or pharmacological overexpression of KLF2 in chondrocytes stimulated with IL-1*β* effectively reduced the rate of apoptosis. Pharmacological inhibition of KLF2 using GGPP resulted in a significant blockade of the KLF2 overexpression-induced effects on apoptosis. Emerging evidence suggests that oxidative stress-derived production of excessive ROS plays a critical role in the progression of OA by inducing chondrocyte apoptosis and ECM degradation [5, 39]. Therefore, we hypothesized that KLF2 might exert a chondroprotective effect by regulating ROS generation. Our results, for the first time, indicate that KLF2 overexpression significantly attenuates IL-1*β*-induced ROS generation in chondrocytes. In summary, these results suggest that KLF2 might be a potential therapeutic target for OA pathogenesis.

Increasing evidence supports the hypothesis that Nrf2 plays a key role in the oxidative stress response during OA progression and activation of Nrf2/ARE signaling is the primary method to reduce the level of ROS [9, 11]. Usually, Nrf2 is retained in the cytoplasm by binding to the cytoskeleton-associated protein Keap1 and is degraded by the proteasome. Upon stimulation, Nrf2 can be released from the Nrf2-Keap1 dimer and translocate to the nucleus where it binds to the AREs present in the promoter regions of target genes, such as HO-1 and NQO1. However, this increase in nuclear translocation of Nrf2 is not enough to offset the oxidative stress induced by OA pathogenesis [11]. Based on this evidence, we hypothesized that KLF2 might exert ECM anticatabolism and antiapoptosis effects by regulating Nrf2 activity. The results of this study indicate that overexpression of KLF2 induces the expression of downstream Nrf2 genes, including HO-1 and NQO1, a phenomenon that was reversed by ML385 (an inhibitor of Nrf2) treatment. Western blot and immunofluorescence analysis revealed that KLF2 overexpression promotes the nuclear translocation of Nrf2, which could explain the KLF2 overexpression-induced increase in HO-1 and NQO1 expression. These results are consistent with those of previous studies showing that KLF2 exerts an antioxidative stress effect by promoting the nuclear translocation of Nrf2 [18, 19, 23]. The translocation of NRF2 is primarily regulated at the level of protein stability in the cytoplasm. Due to continuous ubiquitination and proteasomal degradation, the levels of NRF2 are low at homeostatic status. The major regulator of NRF2 degradation is Kelch-like ECH-associated protein 1 (KEAP1). When KEAP1 binds to the ETGE and the DLG motifs of NRF2, NRF2 can be ubiquitinated by cullin-3- (CUL3-) RBX1/ROC1 ubiquitin ligase and degraded by the 26S proteasome, thus keeping Nrf2 at low levels in the cytoplasm [40]. Another way of NRF2 degradation is mediated by glycogen synthase kinase 3- (GSK-3-) dependent phosphorylation [41]. The degradation of NRF2 also can be regulated by acetylation through histone acetyl transferases p300 [42]. However, at the present study, the underlying mechanism between KLF2 and Nrf2 remains unknown, which should be further explored in the future. One recent study reported that overexpression of KLF2 decreased the levels of H4K8 and H3K9 acetylation [43]. Wu et al. demonstrated that the phosphorylation activity of p38 could be regulated by KLF2 [44]. Furthermore, a previous study highlighted that KLF2 can promote HIF-1*α* degradation through a proteasome-dependent manner [45]. Therefore, these approaches might be the orientations to fully understand the role of KLF2 and Nrf2/ARE signaling in OA. Further research will be required to determine the regulatory mechanism between KLF2 and Nrf2 as a therapeutic target.

To further ascertain that Nrf2 is involved in the chondroprotective effect of KLF2, we inhibited Nrf2 expression using ML385. As expected, the inhibition of MMP13 and apoptosis triggered by KLF2 overexpression was abolished by the inhibition of Nrf2. Taken together, these results demonstrate for the first time that overexpression of KLF2 in chondrocytes results in the inhibition of apoptosis and ECM degradation through Nrf2 activation.

Previously, studies have reported that simvastatin significantly inhibits the IL-1*β*-induced production of matrix-degrading enzymes in *vitro* and protects against the development of cartilage degeneration in OA model mice [46, 47]. It has also been reported that simvastatin triggers the nuclear translocation of Nrf2 in the rat liver [48]. However, the mechanism of simvastatin-mediated protection against the development of OA has not been elucidated in previous studies. In the present study, simvastatin was used as a pharmacological inducer of KLF2 for in *vitro* experiments, and the interesting findings herein are consistent with the findings in these previous reports. Thus, our data indicate that the chondroprotective effect of simvastatin reported in previous studies may be mediated by KLF2.

To further investigate the chondroprotective effect of KLF2, we used an OA animal model to assess the potential therapeutic effects of KLF2 on OA pathogenesis. We induced OA by IA injection of MIA, which has been widely used to investigate OA pathogenesis [49, 50]. Previous studies have extensively demonstrated that adenoviruses can effectively deliver target genes to cartilage tissue [25, 51, 52]. For instance, Son et al. reported that three weekly IA injections of Ad-HSPA1A triggered the effective overexpression of HSPA1A in the cartilage, which clearly abrogated the cartilage erosion caused by OA progression [25]. In the present study, KLF2 overexpression in joint tissues effectively abrogated cartilage degradation caused by IA injection of MIA. Immunohistochemical staining indicated that KLF2 protein levels were significantly decreased in the OA rat tissue. In addition, the adenovirus-mediated overexpression of KLF2 in rat knee joint cartilage downregulated the expression of MMP13. Importantly, we further confirmed that KLF2 overexpression in joint cartilage tissues could effectively promote Nrf2 translocation in *vivo* and Nrf2 inhibition significantly abrogated the KLF2-mediated suppression of apoptosis and MMP13 induced by MIA in *vivo*.

This *in vitro* and *in vivo* study is the first to investigate the chondroprotective effect and mechanisms of KLF2 in OA pathogenesis. However, there were several limitations to this study. On the one hand, the specific regulatory mechanism between KLF2 and Nrf2 remains to be explored, which is the focus of our next study. However, we did not address the involvement of Keap1, which has been identified as an indispensable mediator of the Nrf2-Keap1 redox signaling pathway.

## 5. Conclusions

In summary, our study shows for the first time that KLF2 expression is decreased in OA and closely associated with the severity of OA. KLF2 plays a pivotal role in protecting chondrocytes against IL-1*β*-induced apoptosis and ECM degradation by suppressing ROS generation *in vitro*. Mechanistically, we show that KLF2 overexpression effectively activates Nrf2/ARE signaling to promote the transcription of Nrf2 target genes, hence repressing oxidative stress. Additionally, the overexpression of KLF2 in joint tissues is sufficient to alleviate experimental OA in rats. Our results collectively provide new insights into OA pathogenesis regulated by KLF2 and a rationale for the development of effective intervention strategies for OA.

## Data Availability

The data used to support the findings of this study are available from the corresponding author upon request.

## Supplementary Materials

Supplementary Materials Figure S1: coimmunoprecipitation measuring the interaction of KLF2 and Nrf2.
