# Supplementary figures and images for: KLF2 Protects against Osteoarthritis by Repressing Oxidative Response through Activation of Nrf2/ARE Signaling In Vitro and In Vivo
Source: Oxid Med Cell Longev. 2019 Nov 19;2019:8564681. doi: 10.1155/2019/8564681 (PMC6885785; doi:10.1155/2019/8564681)

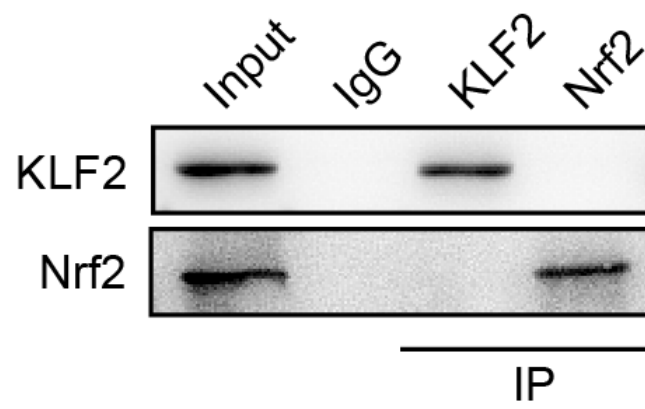

Figure S1: Co-immunoprecipitation measuring the interaction of KLF2 and Nrf2.

Supplement: Supplementary Materials — Figure S1: coimmunoprecipitation measuring the interaction of KLF2 and Nrf2. [file 8564681.f1.pdf]
